# Trend of Gastric Cancer after Bayesian Correction of Misclassification Error in Neighboring Provinces of Iran

**DOI:** 10.31661/gmj.v0i0.1223

**Published:** 2019-07-09

**Authors:** Nastaran Hajizadeh, Ahmad Reza Baghestani, Mohamad Amin Pourhoseingholi, Sara Ashtari, Hadis Najafimehr, Luca Busani, Mohammad Reza Zali

**Affiliations:** ^1^Department of Biostatistics, Faculty of Paramedical Sciences, Shahid Beheshti University of Medical Sciences, Tehran, Iran; ^2^Physiotherapy Research Centre, Department of Biostatistics, Faculty of Paramedical Sciences, Shahid Beheshti University of Medical Sciences, Tehran, Iran; ^3^Basic and Molecular Epidemiology of Gastrointestinal Disorders Research Center, Research Institute for Gastroenterology and Liver Diseases, Shahid Beheshti University of Medical Sciences, Tehran, Iran; ^4^Department of Infectious Diseases, Istituto Superiore di Sanità, Roma, Italy

**Keywords:** Bayesian Analysis, Gastric Cancer, Incidence

## Abstract

**Background:**

Some errors may occur in the disease registry system. One of them is misclassification error in cancer registration. It occurs because some of the patients from deprived provinces travel to their adjacent provinces to receive better healthcare without mentioning their permanent residence. The aim of this study was to re-estimate the incidence of gastric cancer using the Bayesian correction for misclassification across Iranian provinces.

**Materials and Methods:**

Data of gastric cancer incidence were adapted from the Iranian national cancer registration reports from 2004 to 2008. Bayesian analysis was performed to estimate the misclassification rate with a beta prior distribution for misclassification parameter. Parameters of beta distribution were selected according to the expected coverage of new cancer cases in each medical university of the country.

**Results:**

There was a remarkable misclassification with reference to the registration of cancer cases across the provinces of the country. The average estimated misclassification rate was between 15% and 68%, and higher rates were estimated for more deprived provinces.

**Conclusion:**

Misclassification error reduces the accuracy of the registry data, in turn causing underestimation and overestimation in the assessment of the risk of cancer in different areas. In conclusion, correcting the regional misclassification in cancer registry data is essential for discerning high-risk regions and making plans for cancer control and prevention.

## Introduction

Gastric cancer is the fourth most prevalent form of malignancy accounting for 8% of all new cases (989,600 diagnoses) [1]. It is the most prevalent form of cancer in men and the third most common form of cancer (after breast and colorectal cancers) in women in Iran [2]. Its incidence is approximately twice among men as compared to women [3], and over 70% of the cases occur in developing countries [1]. There is a large geographical dispersion in the incidence of gastric cancer on a global scale [3]. Furthermore, there is a large alteration in cancer incidence rate across populations at the lowest and highest risk of gastric cancer [4]. Cancer has been considered as one of the leading causes of death worldwide [5]. It makes population-based and accurate knowledge of cancer occurrence sorely precious to recognize trends and the risk factors that create those trends [6]. The cancer registry data are the main source of data on the burden of cancers by principled registering of the cancer incidence, prevalence, survival, and mortality [7]. Nowadays, their work has expanded into the assessment of cancer screening plans and interventions for cancer control. However, the deficiencies in the registering individuals’ information, including patient’s residence, the primary site of the tumor, date of diagnosis, and date of death [6], make the registered data inaccurate for use in future planning. Most patients prefer to get medical services in the capital of the country or at their neighboring provinces, which are equipped with better medical facilities. Because of the lack of adequate healthcare in their city [8], the patients prefer to register at their neighboring provinces. This is the cause of misclassification. The expected coverage rate of cancer is an indicator of misclassification error in registering cancer incidence, as the expected coverage is reported to be more than 100% in some medical universities and less than 100% in others [9]. Two approaches exist to refine misclassification. The first approach is validating a sample of data by rechecking medical records and expanding its results to the target population [10]. The second approach for correcting the misclassification error is by using the Bayesian method. In this method, the researcher takes prior evidence into account in the analysis [11] by determining prior distribution on the parameters [12]. This study aimed to inquire about the trend of gastric cancer after estimating the misclassification rate in the registry system using the Bayesian method and re-estimating the incidence rate in each province of Iran.

## Materials and Methods

Gastric cancer incidence data from 2004 to 2008 were extracted from the National Cancer Registry (NCR) of Iran, which is published annually by the Ministry of Health (MoH) [9]. The NCR collects cancer incidence data by collaborating with medical universities of the country. Each medical university makes a dataset of new cases of cancer, which are certified by pathology centers. The new cases that are collected are entered into a software that is designed by the MoH. In this stage, duplicate cases are removed, and the remaining recorded cancer cases are coded according to the international coding of disease (10th revision). The MoH sends back the prepared dataset of cancer cases to medical universities. For each medical university, an expected coverage of new cancer cases is calculated, which has been set to 113 per 100,000 population covered by that university. Data were entered into the model in 2 vectors. The first vector contained the age-standardized rate (ASR) for males and females in 4 age groups for the province with less than 100% expected coverage, and the second vector contained the same data for a province with more than 100% of the expected coverage, which is in the neighborhood [13, 14]. Patients were divided into the following 4 groups: those aged 14 years, 15 to 49 years, 50 to 69 years, and more than 70 years. As vectors y1 and y2 contain count data, Poisson distribution was considered for them [15, 16]. For the misclassified parameter (*θ*), which is considered as the probability of recording data in the wrong group, an informative beta prior distribution was assumed. Hence, *θ~beta(a,b)*[17, 18]. Prior values for beta parameters *(a and b)* were selected based on the expected coverage of cancer cases in each province. Expectation of this distribution *a/(a+b)* converges to the misclassification rate. The misclassified parameter is not a known parameter; hence, a latent variable *(U)* was applied as the number of cases that in fact belonged to the first group but were wrongly assigned to the second group. A binomial distribution was assumed for the latent variable, that is, U_i_ | θ,y_1_,y_2_~Binomial(y_i2_,P_i_), and P_i_=(λ_i1_θ)/(λ_i1_θ+λ_i2_), which is the probability of wrong classification in the second group. A sample size of 100,000 is produced from the posterior distribution Beta(∑_i_ U_i_+a,∑_i_ y_i1_+b) by Gibbs sampling [19, 20, 21]. Misclassification rate was estimated by averaging the produced sample from the posterior distribution. Analyses were conducted using the R software version 3.2.0.

## Results

**Figure 1:**
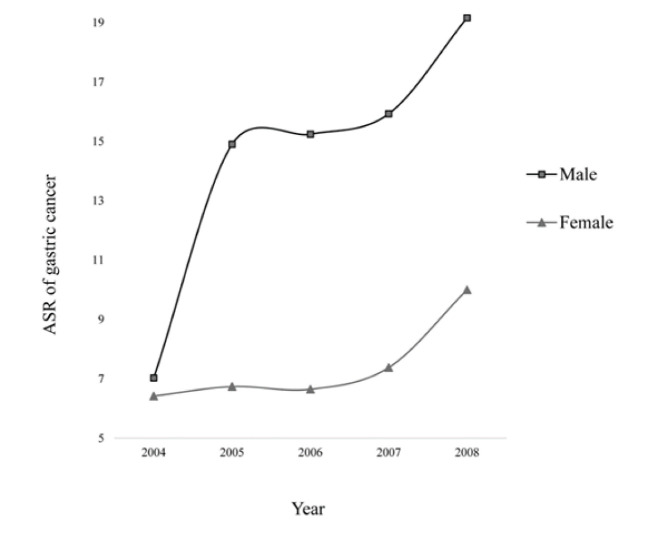
Trend of gastric cancer for two genders (2004 to 2008) in Iran

The registered cases of gastric cancers from 2004 to 2008 in Iran were investigated. The ASR of gastric cancer for females increased from 6.42 per 100,000 populations (1439 persons) in 2004 to 10.00 per 100,000 (2243 persons) in 2008. Similarly, the ASR of gastric cancer for males increased from 7.03 per 100,000 populations (3770 persons) in 2004 to 19.16 per 100,000 (5165 persons) in 2008. The trend of gastric cancer incidence from 2004 to 2008 for both sexes is shown in Figure-1. Among 30 provinces of Iran, the data of 21 provinces were entered into the Bayesian model, two by two. Other nine provinces had a coverage of cancer cases that was almost equal to their expected number of cancer patients; hence, the rates of cancer in those provinces remained unchanged. As an example, the percentage of expected cases for Tehran (the capital of Iran), which is a high-facility province from the perspective of existence of equipped healthcare centers and professional doctors in the central part of the country, was 155.63% in 2008, whereas the Qom, Qazvin, and Markazi provinces that are adjacent to Tehran had just covered 53.9%, 66.3%, and 69.6% of their expected number of new cancer cases, respectively. Thus, Tehran has observed 55.63% more cases than its expected number, and Qom, Qazvin, and Markazi provinces observed fewer cancer cases than their expectation. Expected coverage rates for different provinces of Iran from 2004 to 2008 are based on NCR annuals [9]. After performing the Bayesian analysis, 37% misclassification was estimated between Tehran and Qom, 32% misclassification between Tehran and Qazvin, and 43% misclassification between Tehran and Markazi in 2008. Estimated misclassification rates in other provinces are presented in Table-1. The rate of gastric cancer in the study period, before and after the Bayesian correction of errors, is reported in Table-2.

## Discussion

There was a remarkable misclassification error with respect to the registration of gastric cancer among adjacent areas in Iran. Besides, there was an increase in gastric cancer incidence during the years considered in this study. This increase was higher in males than in females. Highest rates of estimated misclassifications belonged to more deprived provinces such as Sistan, Hormozgan, South Khorasan, North Khorasan, and Bushehr. Also, there was no significant reduction in misclassification rate during the years considered in this study. It indicates that still sufficient effort is not made to prepare healthcare facilities and improve the registration system in all provinces. The well-known risk factors for gastric cancer are *Helicobacter pylori* infections, family history of gastric cancer, and smoking. However, some populations with a high prevalence of *H. pylori* infection and low rates of gastric cancer show that other factors may also be important [3]. Also, the incidence rate among immigrants tends to be similar to those in the country to which they move rather than to those in their country of origin. It can be concluded that environmental factors play a large role in the incidence rates [22, 23]. Thus, it is anticipated that the incidence of cancer is similar in adjacent regions that are exposed to similar circumstances, but there are major differences in the incidence of gastric cancer, which can be justified by misclassification error in recording domicile of patients that causes overestimation or underestimation in the rate of cancer in neighboring areas. Acquiring knowledge about the diffusion of disease among different communities in different areas is an appropriate method for recognizing the factors that influence disease incidence [24] and quantifying the potentials for disease control and prevention [25]. However, usually, spatial analysis is performed based on registered data for finding the geographic pattern of disease and determining high-risk areas. In those types of studies, the existence of misclassification is often ignored. As a result, wrong estimates of risk are achieved in different regions.

## Conclusion

Our study indicates that some misclassification exists in registering cancer incidence. As registered data are the basic source for health policymakers to identify high-risk areas that are in need of more healthcare facilities, misclassification error should be accounted and corrected. Otherwise, it affects the need assessments to dedicate the facilities to the provinces and leads to the allocation of fewer facilities to the provinces that in fact are in need of more healthcare facilities. When valid data are not available, the Bayesian method is a fast and cost-effective way to account for and correct regional misclassification error.

## Acknowledgment

This study was performed inGastroenterology and Liver Diseases Research Center of Shahid Beheshti University of Medical Sciences and supported by grant number 10127.

## Conflict of interest

There is no any conflict of interest regarding the publication of this article.

**Table 1:** Estimated Rate of Misclassification Among Provinces Using Bayesian Method

**Facilitate province**	**Divested province**	**Estimated rate of misclassification**				
		**2004**	**2005**	**2006**	**2007**	**2008**
Razavi khorasan	South khorasan	-	0.28	0.66	0.56	0.43
Tehran	Markazi	0.44	0.32	0.26	0.27	0.43
Razavi khorasan	Sistan	0.73	0.63	0.67	0.65	0.72
Tehran	Qom	0.37	0.23	0.22	0.23	0.37
Tehran	Ghazvin	0.29	0.21	0.22	0.2	0.32
Khozestan	Ilam	0.19	0.51	0.15	0.21	0.26
Khozestan	Bushehr	0.36	0.54	0.44	0.4	0.54
Mazandaran	Golestan	0.46	0.37	0.33	0.32	0.43
Razavi khorasan	North khorasan	-	0.28	0.58	0.48	0.43
Isfahan	Chaharmahal	0.29	0.18	0.21	0.19	0.08
Isfahan	Kohgilouye	0.13	0.18	0.21	0.17	0.08
Fars	Hormozgan	0.69	0.45	0.45	0.52	0.63
East azarbaijan	Ardebil	0.11	0.12	0.11	0.28	0.21
East azarbaijan	West azarbaijan	0.18	0.14	0.13	0.33	0.36

**Table 2:** Age-Standardized Rate of Gastric Cancer Before and After Bayesian Correction

**Provinces**	**Before**					**After**				
	**2004**	**2005**	**2006**	**2007**	**2008**	**2004**	**2005**	**2006**	**2007**	**2008**
**South khorasan**	-	8.38	3.10	4.53	8.66	.	16.11	7.63	10.71	17.66
**Razavikhorasan**	13.46	8.71	15.13	15.12	17.21	11.96	5.76	9.62	9.07	11.61
**Tehran**	6.61	7.80	8.56	7.94	16.50	4.96	6.60	7.53	6.98	14.78
**Markazi**	7.14	6.37	8.91	8.03	8.71	14.39	11.07	13.28	11.80	14.09
**Sistan**	1.98	1.96	2.83	3.11	2.56	7.28	6.49	12.75	13.82	9.95
**Qom**	8.15	9.26	11.05	9.58	10.92	13.82	13.26	14.92	13.19	18.41
**Ghazvin**	11.58	10.46	10.87	11.56	13.10	16.74	13.84	14.22	14.73	19.42
**Khozestan**	5.72	4.08	4.47	5.02	10.80	4.34	1.59	3.18	3.58	8.02
**Ilam**	8.74	5.80	8.77	7.79	11.75	14.57	16.21	12.76	11.75	19.50
**Bushehr**	3.88	3.32	2.27	3.25	3.75	8.79	9.62	5.70	8.24	11.84
**Golestan**	7.99	10.43	12.87	12.33	11.56	15.25	18.04	20.11	19.10	19.20
**Mazandaran**	17.91	16.11	16.81	15.49	22.05	14.75	11.78	12.87	11.66	17.90
**North khorasan**	-	8.23	4.28	6.26	9.00	-	15.72	10.41	12.96	20.12
**Chaharmahal**	5.69	8.83	9.29	12.42	11.03	9.75	12.74	14.96	18.21	13.36
**Isfahan**	6.21	5.69	6.93	7.99	3.25	4.73	3.66	4.91	5.79	2.89
**Kohgilouye**	13.91	9.15	9.52	13.89	11.16	21.47	16.03	16.41	21.87	14.72
**Hormozgan**	2.48	2.96	2.66	3.45	4.37	9.19	8.20	7.43	10.52	18.85
**Fars**	5.91	6.20	5.46	9.25	9.61	4.44	4.61	3.98	6.94	5.06
**Ardebil**	26.61	18.45	18.49	18.52	26.33	31.20	21.92	21.28	26.49	35.11
**East azarbaijan**	10.35	7.49	7.11	19.52	19.35	6.32	4.24	3.92	12.35	11.51
**West azarbaijan**	16.30	15.16	15.41	15.44	12.97	19.88	17.74	18.06	21.61	19.74

## References

[1] Stewart BW, Wild CP. World cancer report 2014. Health 2017.

[2] Ferlay J, Soerjomataram I, Ervik M, Dikshit R, Eser S, Mathers C, et al. GLOBOCAN 2012 v1.0, Cancer Incidence and Mortality Worldwide: IARC Cancer Base No. 11(Internet). Lyon, France: International Agency for Research on Cancer; 2013. Available from: http://globocan.iarc.fr, accessed on day/month/year.

[3] Brenner H, Rothenbacher D, Arndt V (2009). Epidemiology of stomach cancer. Methods MolBiol.

[4] Parkin DM (2006). The global health burden of infection-associated cancers in the year 2002. Int J Cancer.

[5] Arnold M, Karim-Kos HE, Coebergh JW, Byrnes G, Antilla A, Ferlay J (2015). Recent trends in incidence of five common cancers in 26 European countries since 1988: Analysis of the European Cancer Observatory. Eur J Cancer.

[6] Parkin DM (2006). The evolution of the population-based cancer registry. Nat Rev Cancer.

[7] Pourhoseingholi MA, Vahedi M, Moghimi-Dehkordi B, Pourhoseingholi A, Ghafarnejad F, Maserat E (2009). Burden of hospitalization for gastrointestinal tract cancer patients-Results from a cross-sectional study in Tehran. Asian Pac J Cancer Prev.

[8] Mohagheghi MA, Mosavi-Jarrahi A (2010). Review of cancer registration and cancer data in Iran, a historical prospect. Asian Pac J Cancer Prev.

[9] Aghajani H, Etemad K, Gouya M, Ramezani R, Modirian M, Nadali F (2011). Iranian Annual of National Cancer Registration Report 2008-2009 1st ed. Tehran, Tandis.

[10] Lyles RH (2002). A note on estimating crude odds ratios in case–control studies with differentially misclassified exposure. Biometrics.

[11] Corbin M, Haslett S, Pearce N, Maule M, Greenland S (2017). A comparison of sensitivity-specificity imputation, direct imputation and fully Bayesian analysis to adjust for exposure misclassification when validation data are unavailable. Int J Epidemiol.

[12] McInturff P, Johnson WO, Cowling D, Gardner IA (2004). Modelling risk when binary outcomes are subject to error. Stat Med.

[13] Hajizadeh N, Pourhoseingholi MA, Baghestani AR, Abadi A, Zali MR (2017). Bayesian adjustment of gastric cancer mortality rate in the presence of misclassification. World J GastrointestOncol.

[14] Hajizadeh N, Baghestani AR, Pourhoseingholi MA, Ashtari S, Fazeli Z, Vahedi M (2017). Trend of hepatocellular carcinoma incidence after Bayesian correction for misclassified data in Iranian provinces. World J Hepatol.

[15] Pourhoseingholi MA, Faghihzadeh S, Hajizadeh E, Abadi A, Zali MR (2009). Bayesian estimation of colorectal cancer mortality in the presence of misclassification in Iran. Asian Pac J Cancer Prev.

[16] Pourhoseingholi MA, Fazeli Z, Zali MR, Alavian SM (2010). Burden of hepatocellular carcinoma in Iran; Bayesian projection and trend analysis. Asian Pac J Cancer Prev.

[17] Pourhoseingholi MA, Abadi A, Faghihzadeh S, Pourhoseingholi A, Vahedi M, Moghimi-Dehkordi B (2012). Bayesian analysis of esophageal cancer mortality in the presence of misclassification. Ital J Public Health.

[18] Paulino CD, Silva G, Achcar JA (2005). Bayesian analysis of correlated misclassified binary data Comput. Stat Data Anal.

[19] Stamey JD, Seaman JW, Young D (2008). A Bayesian approach to adjust for diagnostic misclassification between two mortality causes in Poisson regression. Stat Med.

[20] Liu Y, Johnson WO, Gold EB, Lasley BL (2004). Bayesian analysis of risk factors for anovulation. Stat Med.

[21] Pourhoseingholi MA (2014). Bayesian adjustment for misclassification in cancer registry data. TranslGastrointest Cancer.

[22] Amoori N, Mahdavi S, Enayatrad M (2016). Epidemiology and trend of stomach cancer mortality in Iran. IJER.

[23] Malekzadeh R, Derakhshan MH, Malekzadeh Z (2009). Gastric cancer in Iran: epidemiology and risk factors. Arch Iran Med.

[24] Mehrabani D, Tabei SZ, Heydari ST, Shamsina SJ, Shokrpour N, Amini M (2008). Cancer occurrence in Fars Province, Southern Iran. Iran Red Crescent Med J.

[25] Zou L, Bao YP, Li N, Dai M, Ma CP, Zhang YZ (2011). Life-style and genital human papillomavirus in a cross-sectional survey in Shanxi Province, China. Asian Pac J Cancer Prev.

